# The Nature of Metastable AA’ Graphite: Low Dimensional Nano- and Single-Crystalline Forms

**DOI:** 10.1038/srep39624

**Published:** 2016-12-21

**Authors:** Jae-Kap Lee, Jin-Gyu Kim, K. P. S. S. Hembram, Yong-Il Kim, Bong-Ki Min, Yeseul Park, Jeon-Kook Lee, Dong Ju Moon, Wooyoung Lee, Sang-Gil Lee, Phillip John

**Affiliations:** 1 Center for Opto-electronic Materials and Devices, Korea Institute of Science and Technology, Seoul 130-650, Korea; 2 Division of Electron Microscopic Research, Korea Basic Science Institute, Daejeon 305-333, Korea; 3 Korea Research Institute of Standards and Science, Daejeon 305-600, Korea; 4 Instrumental Analysis Center, Yeungnam University, Daegu 712-749, Korea; 5 Department of New Materials Science and Engineering, Yonsei University, Seoul 120-749, Korea; 6 Clean Energy Research Center, Korea Institute of Science and Technology, Seoul 130-650, Korea; 7 School of Engineering and Physical Sciences, Heriot-Watt University, Riccarton, Edinburgh EH14 4AS, UK

## Abstract

Over the history of carbon, it is generally acknowledged that Bernal AB stacking of the *sp*^*2*^ carbon layers is the unique crystalline form of graphite. The universal graphite structure is synthesized at 2,600~3,000 °C and exhibits a micro-polycrystalline feature. In this paper, we provide evidence for a metastable form of graphite with an AA’ structure. The non-Bernal AA’ allotrope of graphite is synthesized by the thermal- and plasma-treatment of graphene nanopowders at ~1,500 °C. The formation of AA’ bilayer graphene nuclei facilitates the preferred texture growth and results in single-crystal AA’ graphite in the form of nanoribbons (1D) or microplates (2D) of a few nm in thickness. Kinetically controlled AA’ graphite exhibits unique nano- and single-crystalline feature and shows quasi-linear behavior near the K-point of the electronic band structure resulting in anomalous optical and acoustic phonon behavior.

The structure of graphitic materials including bilayer graphene has been assumed to adopt Bernal AB stacking of graphene planes^1^. This assumption was not unreasonable because AB stacking (interplanar spacing 3.35 Å) is the minimum energy configuration for planar graphene layers and the less stable AA stacking (interplanar spacing 3.53 Å) is not present in pure crystalline graphite^2^. With the progress in fabricating mono- and multilayer graphene^3^, unique Moiré patterns^4^,^5^,^6^,^7^,^8^ are revealed arising from the atomic-resolution transmission electron microscopy (TEM) morphology with rotating angles between graphene planes (i.e., disorderly stacked), and are proved as the signature of disordered graphite. Recently, we have shown that AA’ stacking of graphene planes (where each graphene plane is shifted by 1/2 hexagon from zigzag AA stacking or by 1/4 hexagon from armchair AB stacking) exists, and may be the structure of multi-wall carbon nanotubes (MWNTs) (i.e., helically grown AA’ graphite)^9^,^10^. This AA’ stacking has possibly been interpreted previously as disordered turbostratic structure^11^,^12^,^13^,^14^,^15^ due to the unique X-ray diffraction (XRD) pattern, lacking several peaks for AB graphite (but revealing two peaks at 2θ = 42.4° and 2θ = 77.6°) and further supported by an interlayer spacing of ~3.44 Å^9^,^10^,^11^,^12^,^13^,^14^,^15^. However, MWNTs do not reveal the unique Moiré patterns for disordered graphite, but reveal crystalline features. Indeed, well-developed MWNTs show spot ED pattern^9^,^14^,^15^,^16^,^17^,^18^,^19^ which is the signature of single crystalline. These analysis indicates that AA’ stacking of graphene planes is another crystalline structure of the graphitic phase and the structures of graphitic materials have not been fully appreciated in the literature.

In this paper we present the synthesis of AA’ graphite with two different techniques, (i) thermal-treatment of graphene nanopowders (GNPs), and (ii) plasma seeded growth^10^, where GNPs serve as nuclei (see Methods). We reveal growth kinetics and analytical features as well as electronic and vibrational properties for an AA’ metastable form of graphite.

## Results

**Figure 1:**
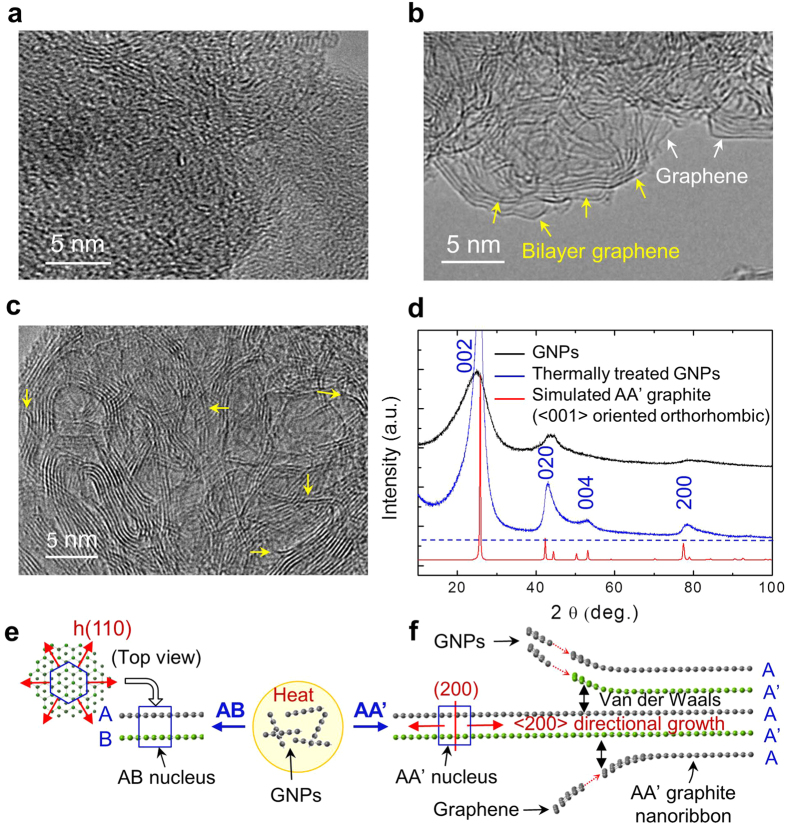
HRTEM images and XRD characterization of GNPs before and after thermal-treatment and growth mechanisms of AB and AA’ graphite. (a) HRTEM image of GNPs. (b,c) HRTEM images of thermally treated GNPs. Bilayer graphene (yellow arrows) prevails together with monolayer pure graphene (white arrows), and mostly appears stacked, resulting in formation of nanoribbon structures. (d) XRD patterns of GNPs before and after thermal-treatment, compared with the simulated pattern for <001> oriented orthorhombic AA’ graphite. (e,f) Growth models of AB (e) and AA’ (f) graphite based on bilayer graphene nuclei. Directional growth of AA’ bilayer graphene nuclei produces 1D single-crystal AA’ graphite. h(110) indicates hexagonal (100) plane of AB structure.

HRTEM images, before (Fig. 1a) and after thermal-treatment (Fig. 1b,c) indicate that GNPs crystallize into graphite in nanoribbon shapes during thermal-treatment. The XRD pattern demonstrates that the graphite nanoribbons are of AA’ stacked graphene planes. The interlayer spacing calculated from the 002 diffraction peak is determined to be 3.44 Å and the pattern fits well with the simulated XRD pattern of <100> oriented orthorhombic AA’ graphite (Fig. 1d). Bilayer graphene prevails in the boundary zone of the TEM sample (yellow arrows in Fig. 1b) as well as in the cross-section of the graphite nanoribbons (yellow arrows in Fig. 1c). The HRTEM and XRD data indicate that the 1D graphite nanoribbon structures are composed of AA’ stacked bilayer graphene.

**Figure 2:**
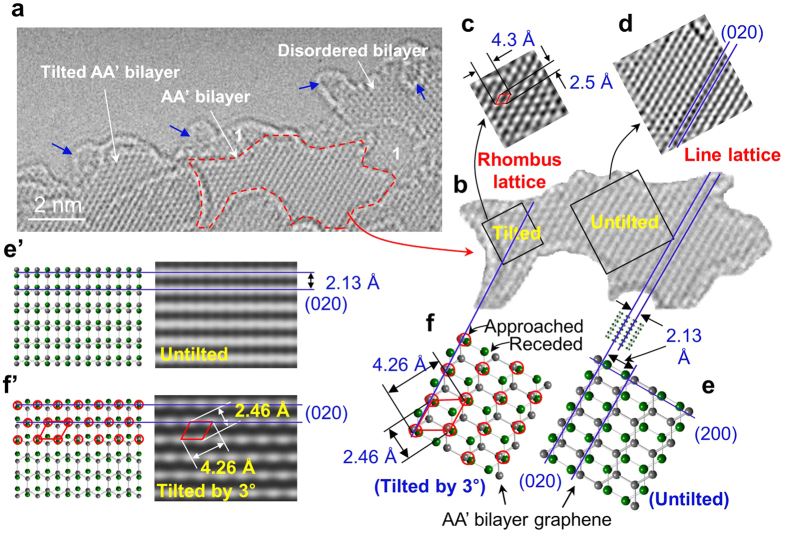
Atomic-resolution TEM morphology imaging AA’ bilayer graphene and its analysis. (a) Atomic-resolution TEM morphology. Monolayer (‘1’) and bilayer graphene (AA’ or disordered) are observed. Blue arrows indicate some particles expected to be related with AB bilayer nucleated. (b) The red dotted domain from a revealing distinct line and rhombus shaped lattice. (c,d) Inverse fast Fourier transform images of the tilted domain (left side) revealing rhombus shaped lattice (c) and untilted domain (right side) revealing line lattices (d). (e,f) Atomic models of AA’ bilayer graphene, untilted (e) and tilted by 3° (f). (e’,f’) Atomic models and JEMS simulation images of AA’ bilayer graphene, untilted (e’) and tilted by 3° (f’). The red circles in (f) and (f’) correspond to white dots in the simulated image (f’).

**Figure 3:**
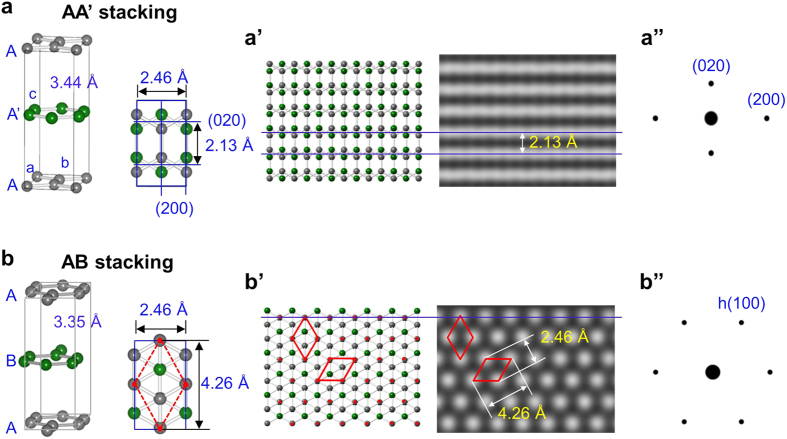
Crystalline models of AA’ and AB graphite and their simulated HRTEM images for bilayer graphene. (a,b) Crystal structures of AA’ graphite (orthorhombic, *Fmmm* #69, *a* = 2.46 Å, *b* = 4.26 Å, *c* = 6.88 Å, α = β = γ = 90°) (a) and AB graphite (b). AB graphite (hexagonal, *p6*_*3*_*/mmc* #194, *a* = *b* = 2.46 Å, *c* = 6.70 Å, α = 60°, β = 120°) was depicted as orthorhombic for simple comparison with that of AA’ graphite. (a’,b’) Atomic models of AA’ and AB bilayer graphene and their simulated HRTEM images. The red dots in (b’) correspond to white dots forming the unique rhombus shaped lattice in the simulation image. (a”,b”) Simulated electron diffraction patterns of AA’ and AB graphite, featured by diagonal and hexagonal pattern, respectively. h(100) represents hexagonal (100) plane of AB structure.

Atomic-resolution TEM image for the thermally treated GNPs, AA’ graphite nanoribbons, reveals the atomic lattices of AA’ bilayer graphene as shown in Fig. 2. From their edge structures^10^, the number of overlapped graphene layers can be identified (Fig. 2a) and mono- or bilayer graphene is evident. Bilayer graphene images reveal distinctive line lattices (except for the domain showing disordered bilayer graphene), where interline distance is 2.13 Å (Fig. 2b). The line lattices are identical to simulated images for AA’ bilayer graphene and are assigned to (020) planes (Fig. 3a’). The prominent (020) planes exhibit a d-spacing of 2.13 Å (Fig. 2e’). The red dotted bilayer graphene domain comprises the rhombus shaped lattice whose widths are about 4.3 Å and 2.5 Å (Fig. 2c) and are adjudged to be ‘tilted’ AA’ bilayer graphene (Fig. 2f,f’). When AA’ bilayer graphene is tilted by a few degrees, the original symmetry is broken due to the unique overlapping of AA’; the two atoms belonging to alternate pair on the (020) planes are fractionally closer (red circles in Fig. 2f) while those of the other pair of atoms recede (unmarked in Fig. 2f). Simulations confirm that such structures can generate rhombus shaped lattice with enhanced intensity due to the former pair of atoms. Thus, we attribute the rhombus shaped lattice to AA’ bilayer graphene tilted by about 3° on the TEM stage (Fig. S1a). The rhombus shaped lattice (Fig. 2c) is comparable with the rhombus lattice of AB Bernal graphite prevailing in the literature^20^,^21^,^22^,^23^,^24^,^25^,^26^, where each white dot of the AB rhombus shaped lattice is due to amplification from the two overlapped atoms of the AB bilayer graphene (Fig. 3b).

**Figure 4:**
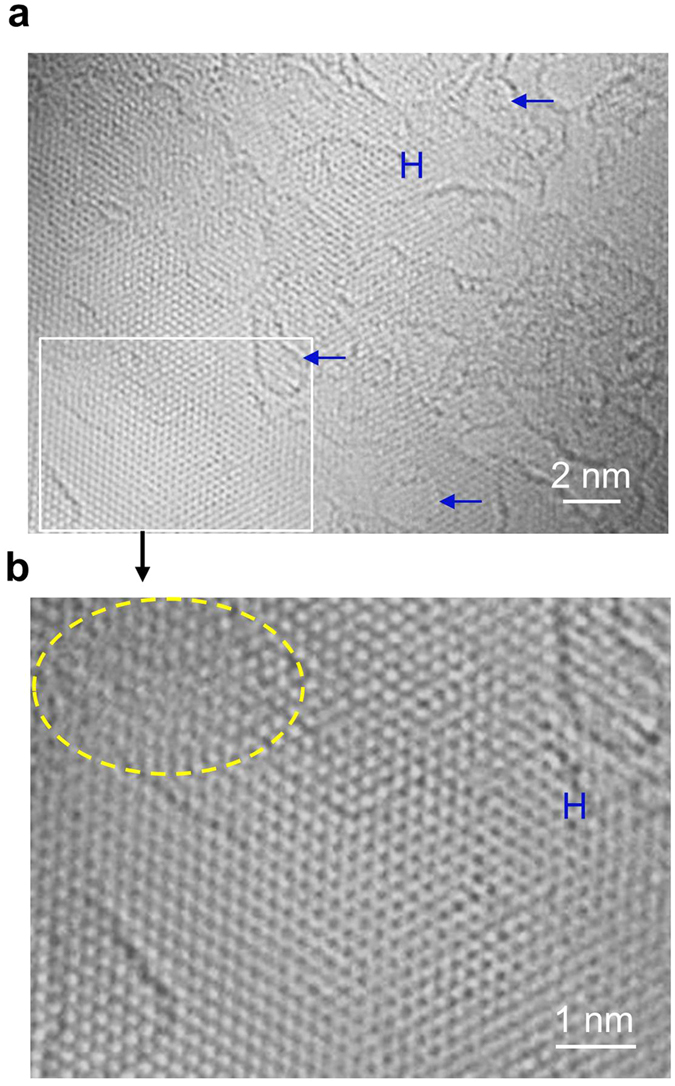
Atomic-resolution TEM morphology imaging overlapped AA’ bilayer graphene. (a) Atomic-resolution TEM morphology imaging the inner zone of the TEM sample where AA’ bi- or multilayer graphene samples appear to be overlapped during TEM sampling. (b) Magnified image of the rectangular zone in (a) revealing the unique line, rhombus, hexagonal (indicated by ‘H’), and disordered (ellipse) lattices. Blue arrows indicate some particles expected to be related with AB bilayer nucleated.

The rhombus shaped lattice for tilted AA’ bilayer graphene prevails in the inner zone of the TEM sample, as shown in Fig. 4, where the graphene layers appear to be overlapped with higher tilt angles compared with the boundary zone of the samples. Some domains reveal the unique rhombus shaped lattice for AA’ bilayer graphene, which is tilted by 12° on the TEM stage (Fig. S1a), and the pattern of distinct dots appears similar those of untilted AB bilayer graphene (Fig. 3b). The line lattices for (untilted) AA’ bilayer graphene are also observed with hexagonal lattices (‘H’ in Fig. 4). Hexagonal symmetry lattices may be obtained from stacked graphene layers, because multilayer graphene (graphite) also yields the hexagonal lattices coupled with defocusing (Fig. 7 of ref. 24). Similarly high-resolution TEM (HRTEM) simulations confirm that the hexagonal lattices appear from AA, AB as well as AA’ bilayer graphene with defocusing (Fig. S1). We attribute the irregular lattices in yellow ellipse of Fig. 4b to AA’ bilayer graphene based disordered stacking, which are relatively normal compared with those of AA or AB bilayer graphene. On the other hand, overlapped morphology of AA’ bilayer graphene does not reveal the unique hexagonal Moiré patterns (Fig. S2), thus is unique compared with those of AA or AB bilayer graphene. This lack of the Moiré patterns can be the signature of AA’ stacking as supported by HRTEM simulations.

**Figure 5:**
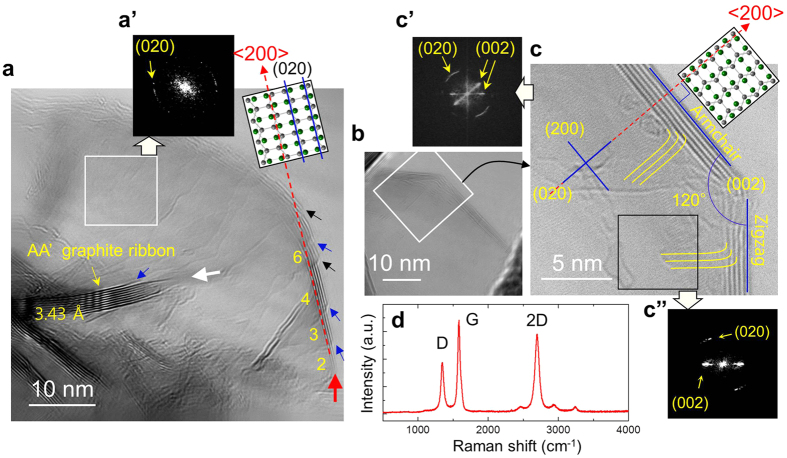
Single-crystal AA’ graphite plates grown under high density plasma condition generated at 200 Torr of pressure. (a) HRTEM image of single-crystal AA’ graphite plate (~2 μm^2^ in area). Number of graphene layers is indicated by yellow color. (a’) FFT pattern for the rectangular in (a). (b,c) A well-developed single-crystal AA’ graphite plate with a size of ~1 μm^2^ in area. (c’,c”) FFT patterns for the whole and rectangular zone, respectively. (d) A Raman spectrum obtained from the samples.

The single-crystalline features with AA’ stacking are evident in the samples prepared by the plasma seeded growth as shown in Fig. 5. The unique HRTEM image in Fig. 5a, revealing a thin graphite plate comprising 2~10 graphene layers locally (~3 nm in thickness), indicates that the initial bilayer graphene (thick red arrow) have merged mono- (black arrow) or bilayer (blue arrow) graphene during the growth, resulting in the development of big single-crystal. The sequence of merging layers gives rise to stepwise growth from bilayer graphene (thick red arrow) to the thin graphite microplate (Fig. 5a). FFT pattern, shown in Fig. 5a’, which is clearly different from hexagonal spots of single-crystal AB graphite^27^ (Fig. 3b”), demonstrates that the thin single-crystal is of AA’ stacking. The graphite ribbon with a tail (white arrow) reveals interplanar spacing of 3.43 Å, indicating that it is also resulted from the unique stepwise growth of AA’ bilayer graphene. HRTEM images, shown in Fig. 5b,c, provide clear evidence that the thin graphite microplate comprising 6 graphene layers (~2 nm in thickness) consists of a AA’ single-crystal, which is demonstrated by the FFT patterns (Fig. 5c’ and c”). We attribute the elongated (020) signals to curved ends of the plate (yellow lines) which are supported by the revelation of (002) layers at both edges of the plate. The curvature can make the graphene sheets tilt, thus break the symmetry locally as confirmed in Fig. 2, explaining the appearance of the elongated (020) signals. The spot signals of (020) from the square zone where the curved end part is excluded (Fig. 5c”) supports our analysis. From the crystallographic relationship of AA’ structure (Fig. 5a), we infer that the crystal is resulted from <200> preferred growth of AA’ bilayer graphene. Raman spectrum reveals sharp and strong G (~1584 cm^−1^) and 2D (~2699 cm^−1^) peaks, indicating high crystallinity of AA’ graphite. We attribute the appearance of clear D peak (~1348 cm^−1^) to nanoribbon structures (with large area of edge) of AA’ graphite.

From the analysis of AA’ bilayer graphene and the appearance of the graphitic nanoribbon or microplate, we infer the formation mechanism of AA’ graphite, as depicted in Fig. 1f. AA’ bilayer graphene nuclei generated from unstable GNPs grow in competition with other types of graphene stacking albeit mostly with stable AB bilayer graphene nuclei. With <200> texture growth induced by the unique anisotropic overlapping of AA’ stacking, the nuclei dominate the growth and become dimensionally larger with incorporation of graphene or other AA’ bilayer graphene during the growth, resulting in formation of single-crystal AA’ graphite nanoribbons or microplate.

**Figure 6:**
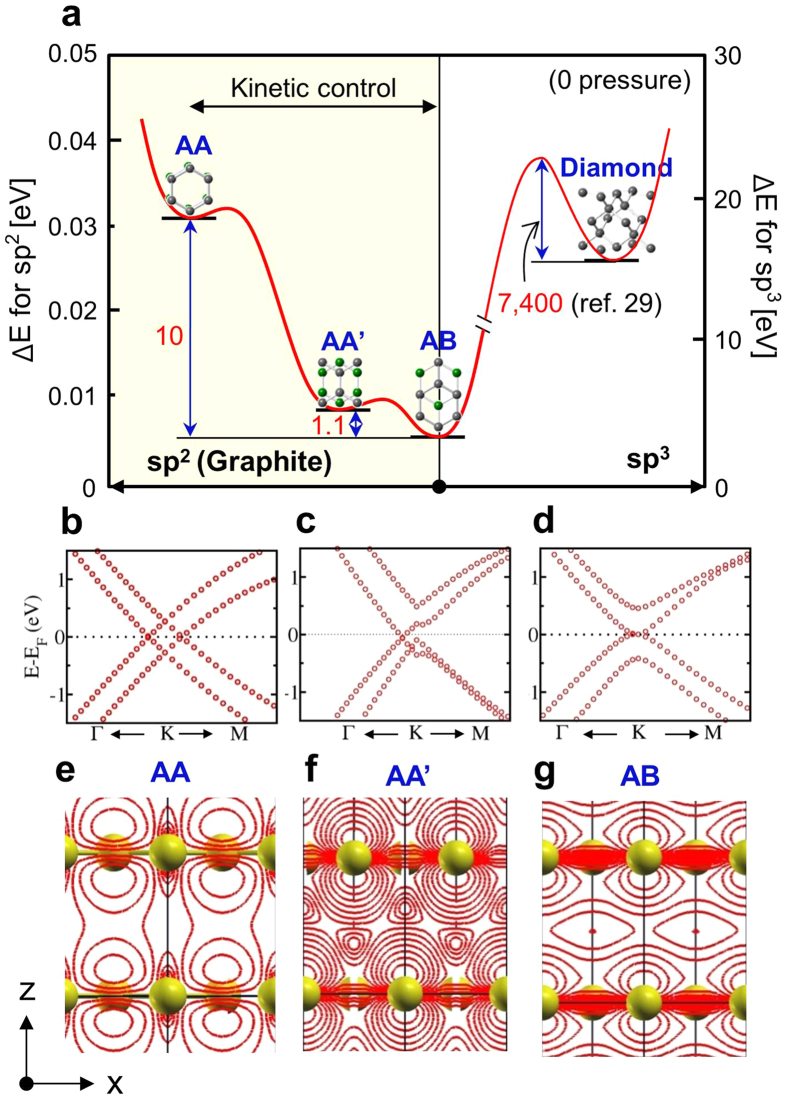
Energy landscape of *sp*^*2*^ and *sp*^*3*^ carbon allotropes and electronic properties of bilayer graphene. (a) Energy landscape of various bilayer graphene, compared with diamond. (b–d) Electronic band structure of AA, AA’ and AB bilayer graphene. (e–g) Iso-charge contour plot for AA, AA’ and AB bilayer graphene, revealing 2D charge density. Transition energy from AA’ to AB bilayer graphene is expected to be very little compared with the barrier energy. Unit for energy is meV/atom.

It might be expected that AB bilayer graphene may exist in the sample. The starting materials in this study are GNPs, which possess AB stacking and serve as nuclei of AB bilayer graphene. We anticipate that the ~1 nm sized circular patches in Fig. 2a (blue arrows) are due to nucleated AB bilayer graphene but further growth ceases (Fig. 1e) due to the preferred texture growth of AA’ bilayer graphene (Fig. 1f). In fact, the crystallization from GNPs at 1,500 °C may depend on kinetics of the conversion of AA, AB and AA’ bilayer graphene because the disordered GNPs exist in a thermodynamically unstable state (Fig. 6a). This is normally masked by the fact that graphite (AB graphite) is generally crystallized at the temperature range of 2,600~3,000 °C^28^ where kinetic considerations are dominated by thermodynamic. Stacking structures at temperatures below 2,600 °C may be determined by the migration kinetics of the graphene planes. The analysis is supported by energy consideration shown in Fig. 6a. AA’ stacking exists in a local potential well in the energy landscape, although AB stacking is the lowest energy configuration. Thus we defined AA’ stacking as a kinetically stable structure of graphite, similar to the case of diamond^29^.

**Figure 7:**
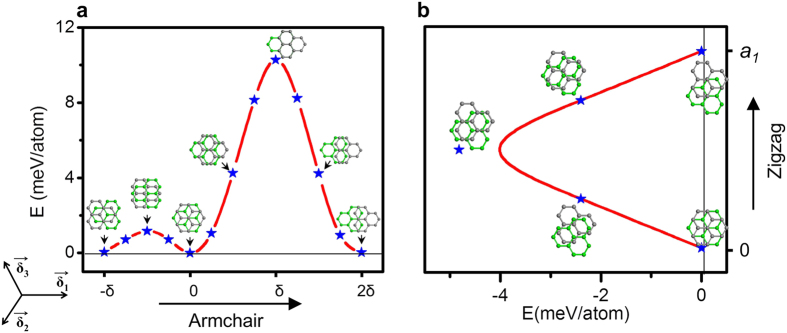
Energy landscape for the sliding of one layer with respect to another layer for bilayer graphene. (a) Armchair direction. (b) Zigzag direction. It is clearly sheen that AB stacking is most stable. However AA’ stacking can be envisaged with possessing little high energy. AA stacking possess much higher in energy among all the structures. δ in (a) and *a*_*1*_ in (b) are 1.42 Å and 2.46 Å (lattice constant of hexagonal AB crystal), respectively.

To unravel the features of stacking, we simply model them using bilayer graphene system. In the bilayer graphene, sliding of top graphene layer with respect to bottom layer gives rise to various kinds of stacking sequences (Fig. 7). The relative sliding can be modeled as V(*u*) = A. W. [1 − cos (2π*u*)], where A is the constant, W is the barrier height and *u* is the displacement of the sliding vector. The barrier height is 1.1 meV/atom during traversing along −1/2*δ* direction (*δ*, sliding along armchair direction), and this qualitatively matches with the result of Popov *et al*.^30^. This −1/2*δ* sliding is equivalent to half the bond length of C-C atoms along armchair direction, giving rise to AA’ stacking. Contrary, traversing along +*δ* gives rise to AA stacking which possess the barrier height of 10 meV/atom and ±*a*_*1*_ along zigzag direction to AB stacking with barrier height of 5 meV/atom.

The consequence of stacking features is reflected at the energy dispersive electronic structure diagram (Fig. 6b–d) (also see Fig. S3). The interlayer coupling changes the linear band to parabolic band for AB stacking. For the AA stacking, the degeneracy of the Dirac cone is symmetrically lifted with +ve and −ve shifts in the momentum axis^31^. The degeneracy is lifted asymmetrically for AA’ stacking. The AA’ stacking shifts the K points not only in the momentum axis, but also in the energy axis, thereby opening the gap of ~0.35 eV (0.00 and 0.50 eV for AB and AA stacking, respectively). The qualitative distinct features reveal the signature of the different stacking structure implying the consequences of the governing physical phenomena.

The origin of the above features can be explained by observing the electron clouds of the π orbitals. The localized π orbitals are out of phase for AB stacking, and are in phase for the AA stacking. But for the AA’ stacking they are in between. Hence the interaction of the π-π electron clouds tries to maintain at their equilibrium for the AA’ stacking which is in between AB and AA stacking. Figure 6e–g shows the iso-charge contour plot on XZ plane which is direct indication of arrangement of localized orbitals for AB, AA and AA’ stacking (Fig. S4). The special features from AA’ are the key messages and these finding distinct AA’ stacking from the conventional AB and AA stacking.

## Discussion

AA’ graphite appears as single-crystal nanoribbon or microplates of a few nm in thickness, and thus reveals unique XRD pattern for textured AA’ graphite with the interlayer spacing of around 3.44 Å and ED pattern for the orthorhombic crystal. AA’ graphite reveals distinctive line lattices, but does not reveal the unique hexagonal Moiré patterns. AA’ stacking, a relative metastable system, exhibits distinct electronic properties and anomalous vibrational properties, which makes it unique compared to AA and AB stacked graphite. Our results provide fundamental understanding on the structures of graphene based materials and a route to fabricating designable low dimensional single-crystal graphite at the relatively low temperature of ~1,500 °C.

## Methods

We synthesized AA’ bilayer graphene based structures by two methods, (i) thermal-treatment of GNPs, and (ii) plasma seeded growth of GNPs^10^. The GNPs with a size of ~5 nm were prepared by ball-milling commercial MWNTs (CM-95, 95 wt% purity, Hanwha nanotech) and subsequent purification of the milled sample. Then 1 g of GNPs was subjected to thermal-treatment at 1,500 °C for 30 minutes in a vacuum chamber (10^−5^ Torr). Thermally treated samples were analyzed by an XRD (Rigaku D/MAX 2200 V diffractometer using a Cu*K*_*α*_ source) and two HRTEM equipment, i) JEM-2100F operating at 200 kV and ii) Cubed with and a monochrometer operating at 80 kV. Plasma seeded growth was performed under direct current plasma generated at 200 Torr and temperature was kept at 1,000 °C. Seeded grown graphene samples were analysed by an aberration corrected energy-filtered TEM (200 kV, Libra 200 HT Mc Cs TEM; Carl Zeiss) and Raman (Renishaw In-Via Raman Microscope with laser excitation of 532 nm and spot size of 1–2 μm). The growth condition is similar to that of diamond growth^10^. On this procedure GNRs serve as nuclei. We simulated HRTEM morphologies for AA, AB, and AA’ bilayer graphene using JEMS computer software^32^ at different defocusing from 0 and 25 nm. Simulation parameters were based on FEI Titan TEM systems. An 80 kV accelerating voltage was used like the case of experimental imaging to reduce sample damage. Effects of tilt angle up to 12° on image pattern were evaluated for AA’ bilayer graphene (Fig. S1). Our calculations are based on first-principles density functional theory (DFT) as implemented in the QUANTUM ESPRESSO simulation package^33^. Generalized gradient approximation (GGA) was used for exchange correlation energy of electrons and ultra-soft pseudo potentials to represent interaction between ionic cores and valence electrons^34^,^35^ Kohn-Sham wave functions were represented with a plane-wave basis with an energy cutoff of 40 Ry and a charge density with a cutoff 240 Ry. Integration over irreducible Brillouin zone for charge density and total energy was performed with a uniform mesh of 48 × 48 × 1 mesh of *k* points^36^. Occupation numbers were smeared using Methfessel-Paxton scheme^37^ with broadening of 0.01 Ry. Errors in the stresses and total energy due to basis-set size, smearing parameter, and *k* points are converged to less than 0.03 GPa and 10^−8^ Ry, respectively. Dynamical matrices in Brillouin zone were computed with a grid of 4 × 4 × 1 using perturbative linear response approach used in DFT.

## Additional Information

**How to cite this article**: Lee, J.-K. *et al*. The Nature of Metastable AA’ Graphite: Low Dimensional Nano- and Single-Crystalline Forms. *Sci. Rep.* **6**, 39624; doi: 10.1038/srep39624 (2016).

**Publisher's note:** Springer Nature remains neutral with regard to jurisdictional claims in published maps and institutional affiliations.

## Supplementary Material

Supplementary Information
