# Supplementary material for: The Nature of Metastable AA’ Graphite: Low Dimensional Nano- and Single-Crystalline Forms
Source: Sci Rep. 2016 Dec 21;6:39624. doi: 10.1038/srep39624 (PMC5175192; doi:10.1038/srep39624)
Supplement: Supplementary Information [file srep39624-s1.pdf]

Supplementary Information for

The Nature of Metastable AA' Graphite: Low Dimensional Nano- and Single-Crystalline Forms

Jae-Kap Lee,[1*] Jin-Gyu Kim,[2] K. P. S. S. Hembram,[1] Yong-Il Kim,[3] Bong-Ki Min,[4] Yeseul Park,[1,5] Jeon-Kook Lee,[1] Dong Ju Moon,[6] Wooyoung Lee,[5] Sang-Gil Lee,[2] and Phillip John[7]

[1]Center for Opto-electronic Materials and Devices, Korea Institute of Science and Technology, Seoul 130-650, Korea

[2]Division of Electron Microscopic Research, Korea Basic Science Institute, Daejeon 305-333, Korea

[3]Korea Research Institute of Standards and Science, Daejeon 305-600, Korea

[4]Instrumental Analysis Center, Yeungnam University, Daegu 712-749, Korea

[5]Department of New Materials Science and Engineering, Yonsei University, Seoul 120-749, Korea

[6]Clean Energy Research Center, Korea Institute of Science and Technology, Seoul 130-650, Korea

[7]School of Engineering and Physical Sciences, Heriot-Watt University, Riccarton, Edinburgh EH14 4AS, UK

***Corresponding Author**

E-mail: jklee@kist.re.kr

CONTENTS

- Figure S1-S4
- Explanations for Figure S3 and S4
- References

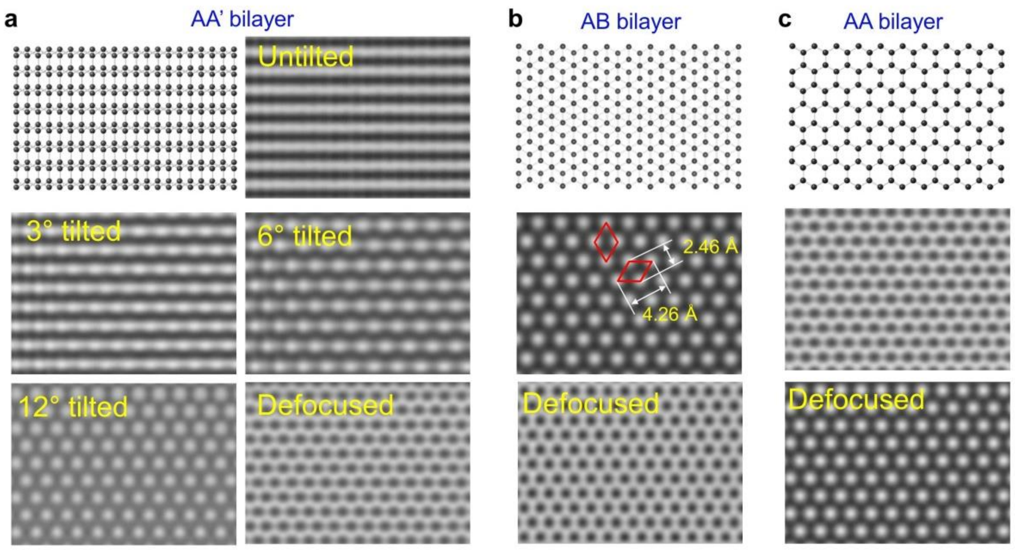

Figure S1. HRTEM simulations. (a) Simulated images for AA' bilayer graphene with various tilt angles. (b,c) Simulated images for untilted AB and AA bilayer graphene. Data confirm that plane view atomic-resolution TEM morphology of stacked graphene layers (i.e., graphite) varies with tilt angles of sample on TEM stages as well as defocusing. It is evident that the hexagonal lattices of carbon atoms appear from AA, AB as well as AA' bilayer graphene with defocusing.

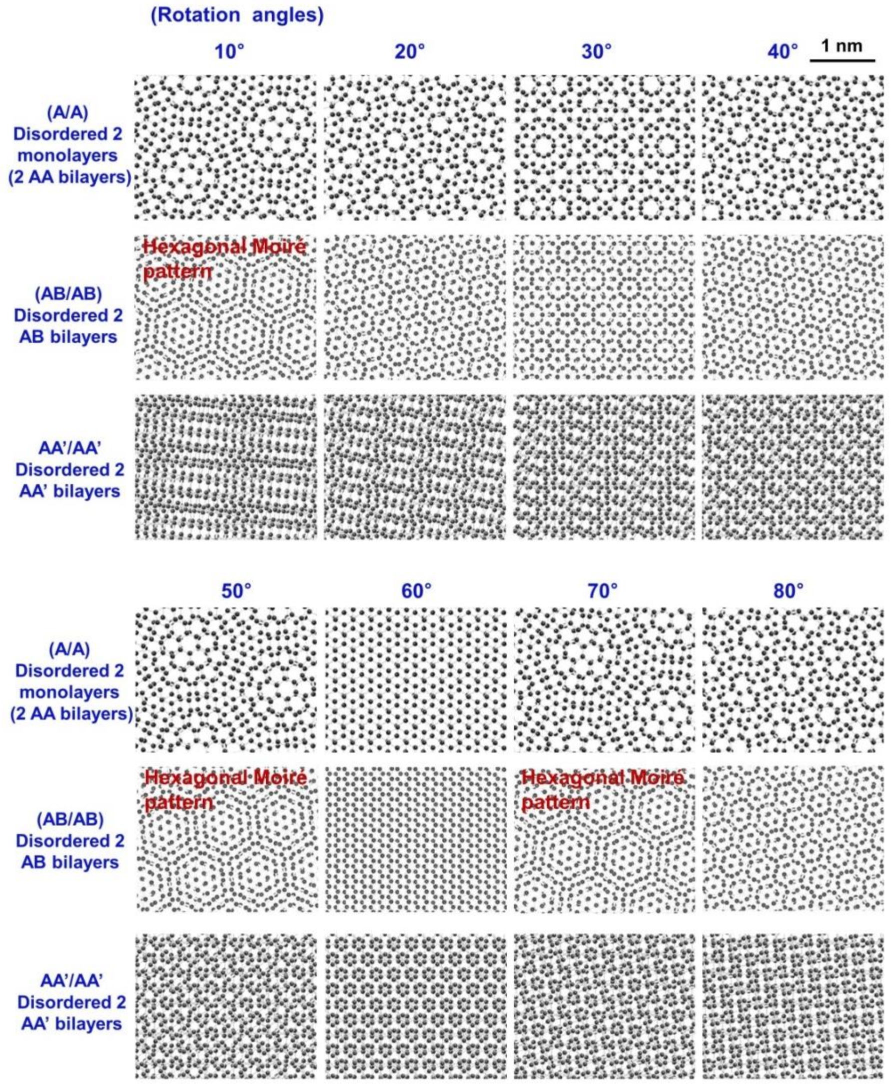

Figure S2. Overlapping (disordered) morphology series for AA, AB and AA' bilayer graphene misoriented with respect to each other. Morphology for the disordered graphene layers varies with the misoriented angles. The unique hexagonal Moiré patterns appear only from disorderly stacked AB/AB series. The morphologies of disordered AA'/AA' series are relatively normal compared with those of AA/AA or AB/AB series, explaining the atomic-resolution TEM morphology in fig. 4.

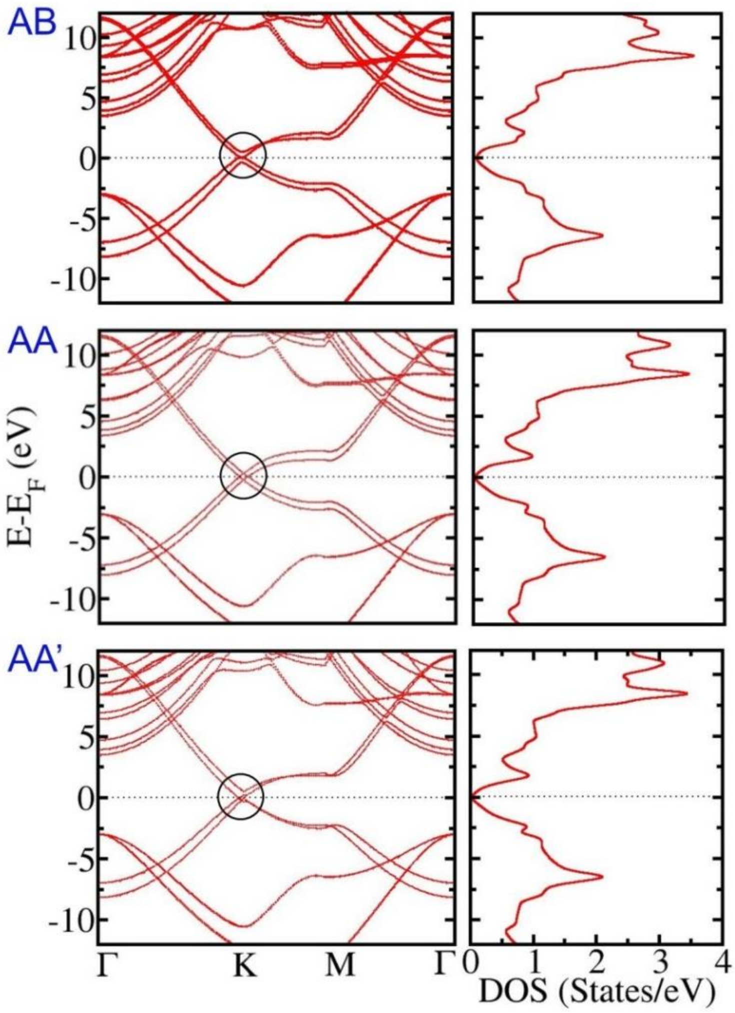

Figure S3. Electronic band structures and electronic density of states of AB, AA and AA' stacking. Encircled portions are enlarged and shown in Figure 4.

Figure S3 shows the full electronic band structure and electronic density of states of AB, AA and AA' stacking. The significant distinct features are observed near the K point. For AB staking the Dirac cone is changes to parabolic band. For AA stacking the Dirac cones are left and right shifted in the momentum axis. For AA' the signature gets more complicated. The special point not only shifted in the momentum axis, but also in the energy axis asymmetrically. The consequences of the interlayer coupling through *2pz* orbitals are manifested with the signature of band shifting at the K point, for different stacking.

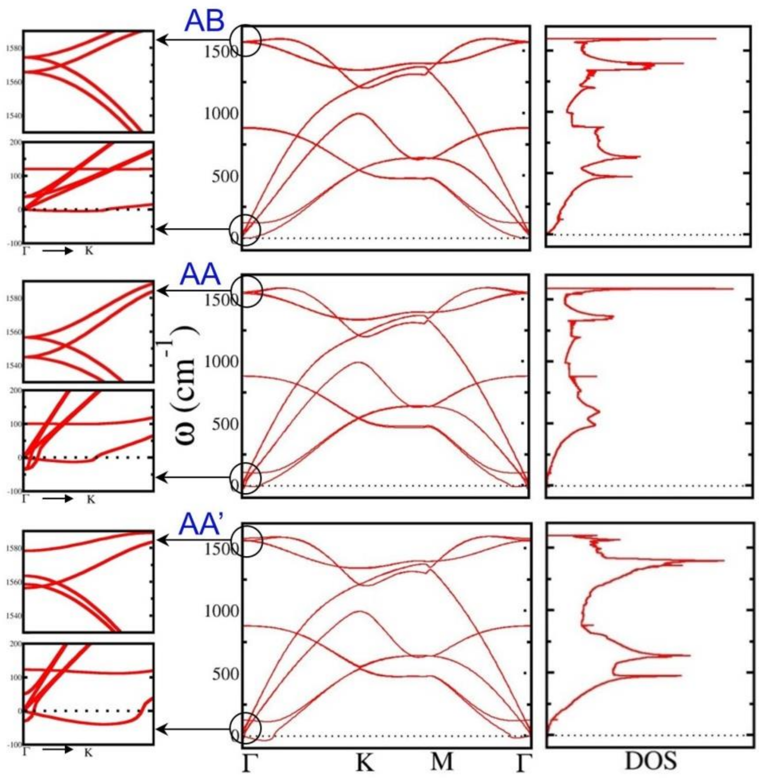

Figure S4. Phonon band structures and phonon density of states of AB, AA and AA' stacking.

Figure S4 shows the full phonon band structure and phonon density of states of AB, AA and AA' stacking. The absence of imaginary frequencies for AB stacking shows the stability of the structure. However little imaginary frequency for AA and AA' shows the less stable structure compared to that of AB. However they are possible with little sliding, those possess small extra energy.

Viewing the vibrational features of the above structures, we reveal the difference in the spectroscopic signature. The interactions between planar carbon atoms are stronger than between inter planar carbon atoms. Hence their effect on vibrational properties is relatively small, but meaningful.

AA' stacking shows anomalous longitudinal-optic transverse-optic (LO-TO) and longitudinal acoustic transverse acoustic (LA-TA) behaviors which are different from those of AB and AA stacking. For instance, there is no LO-TO splitting for AB and AA stacking. For AA stacking, the degenerate bands are softer than AB stacking by ~20 cm^{-1}. But for AA' stacking the complex anomalous behavior arises. For one band, the LO-TO band is degenerate little away from the Γ point, but the band show finite splitting of ~15 cm^{-1} at Γ point. Similar behavior is observed by Choi *et al*. while sliding one layer over another in armchair direction[1].

Supplementary Information References

1. Choi, S.-M., Jhi, S.-H. & Son, Y.-W. Anomalous optical phonon splittings in sliding bilayer graphene. *ACS Nano* **7,** 7151-7156 (2013).
